# Psychiatric and somatic health of homeless individuals in the context of their migration history

**DOI:** 10.1093/eurpub/ckac131.514

**Published:** 2022-10-25

**Authors:** F Bertram, A Hajek, H-H König, B Wulff, B Ondruschka, K Püschel, F Heinrich

**Affiliations:** 1 Institute of Legal Medicine, University Medical Center Hamburg-Eppendorf, Hamburg, Germany; 2 Health Economics and Health Care Research, University Medical Center Hamburg-Eppendorf, Hamburg, Germany

## Abstract

**Background:**

Descriptions of homeless individuals’ somatic and psychiatric health status remain scarce. The heterogenicity of the population is increasing, with more people migrating within the European Union. Migration history has been described as a determinant of health and healthcare access.

**Methods:**

A multicenter cross-sectional study design included homeless individuals in Germany. Using interview-based questionnaires, the prevalence of mental and somatic illnesses, as well as healthcare use and access, were determined. Multinominal logistic regression analysis was performed to examine the influence of the homeless migration history on health status.

**Results:**

306/635 (48.2%) of the homeless individuals were born outside Germany; 213/306 (69.6%) came from another EU country. Homeless people from EU countries frequently reported economic reasons for leaving their home country (51.0%) and entering Germany (64.4%). Compared to homeless individuals of German origin and homeless non-EU migrants, they stated to live rough (48.2% p = 0,03), not hold health insurance (62.4% p < 0,0001), and not receive state funds (82.6% p < 0,0001) more often. Prevalences of psychiatric and somatic illnesses among homeless people were high compared to the general German population. There were no differences observed between the prevalence of chronic diseases if stratified by the origin of the homeless individuals.

**Conclusions:**

Homeless individuals report higher prevalences of psychiatric and somatic illnesses than the general population. Compared to homeless people of other origins, homeless EU migrants may be disadvantaged in their housing situation and integration into the German social security system.

**Key messages:**

• Programs aiming to integrate homeless people into mainstream health care should focus on homeless EU migrants.

• Our data underline the need for specific care services for homeless individuals.

